# Evaluation of the California American Academy of Pediatrics Resident Advocacy Rotation: A State-Wide Collaborative Approach to Teaching Advocacy to Pediatric Trainees

**DOI:** 10.7759/cureus.117495

**Published:** 2026-10-05

**Authors:** Vivien K Sun, Cambria Garell, Susan Wu

**Affiliations:** 1 Department of Pediatrics, Stanford University School of Medicine, Palo Alto, USA; 2 Department of Pediatrics, University of California, Los Angeles David Geffen School of Medicine, Los Angeles, USA; 3 Department of Pediatrics, University of Southern California Keck School of Medicine, Los Angeles, USA

**Keywords:** child advocacy, community pediatrics, education collaborative, medical education, virtual learning

## Abstract

Background

In 2021, the California Collaborative in Community Pediatrics and Legislative Advocacy Training and California’s American Academy of Pediatrics co-developed the Resident Advocacy Rotation (RAR). This twice-monthly virtual workshop for pediatric trainees focuses on child health policy and advocacy skills, taught by faculty from across the state. We describe trainee and faculty satisfaction and perceived impact after the first three years of the RAR.

Methodology

Between July 2021 and June 2024, all trainees who participated in RAR were invited to complete a satisfaction survey immediately following the session. Faculty who facilitated RAR sessions or led Community Pediatrics/Advocacy rotations were invited to complete a satisfaction survey between January and May 2024. Multiple-choice questions in the trainee and faculty surveys were analyzed using descriptive statistics. Open-ended questions were analyzed inductively using conventional content analysis.

Results

A total of 67 RAR sessions were delivered, with 312/721 (43%) registered trainees from 13 residency programs completing the survey. Trainees agreed that RAR included content they hoped to gain, effectively taught advocacy skills, and introduced new ideas. Trainees highlighted the value of learning about advocacy resources, skills, and collaborating with trainees from other programs. Overall, 17 of 25 (68%) faculty facilitators completed the survey, representing 10 residencies. Faculty identified passion for teaching, collaborating across the state, partnering with community organizations, and growing their own skills and career as the most rewarding aspects of participating in RAR.

Conclusions

RAR was viewed favorably by both trainees and faculty facilitators, suggesting that a collaborative statewide curriculum is a well-received and feasible approach to advocacy education.

## Introduction

Physician advocacy is defined as an action “to promote social, economic, educational, and political changes that ameliorate the suffering and threats to human health and well-being” [1]. Advocacy skills have been increasingly emphasized during physician training programs, including medical school and residency [2,3]. In pediatrics, the Accreditation Council for Graduate Medical Education (ACGME) has required training in community pediatrics and child advocacy for residency programs since 2001, recognizing these as core physician skills [4].

Despite the increasing prominence of advocacy in graduate medical education, residency program directors have cited limited faculty expertise, time, curricular flexibility, and resources as barriers to the development of advocacy curricula [5]. Advocacy consortiums pooling education resources across a region have been leveraged to address many of these barriers. For example, the California Collaborative in Community Pediatrics and Legislative Advocacy Training (“California Collaborative”) was established in 2007 to share advocacy-related educational resources and faculty development statewide [6]. Participation in the California Collaborative increased the number of pediatric training programs adopting components of advocacy curricula such as letter-writing campaigns and contacting legislators [6]. Similarly, the Carolinas Collaborative for Child Advocacy and Resident Education developed the “Advocacy Training Essentials Toolkit” to help programs enhance their educational curricula [7], and showed that involvement in the multi-state collaborative improved the quality of advocacy education for trainees across the region [8]. While advocacy collaboratives have pooled curricular and faculty development resources, few have offered synchronous, recurrent advocacy teaching on a statewide scale [9-12]. According to the Community Pediatrics Training Initiative, there are currently nine regional pediatric advocacy collaboratives in the United States [13].

In 2021, the California Collaborative and California’s American Academy of Pediatrics (AAP-CA) co-developed the Resident Advocacy Rotation (RAR), a twice-monthly virtual workshop for pediatric trainees focused on child health policy and advocacy skills. The primary objective of this study was to describe pediatric trainees’ satisfaction with the RAR over its first three years, including perceived relevance, applicability, and the aspects trainees valued most. The secondary objective was to describe faculty facilitators’ satisfaction, including perceived value to themselves and their institutions, willingness to facilitate again, rewarding and less rewarding aspects of teaching, and perceived effects on career development.

This article was previously presented as a meeting abstract at the 2025 Pediatric Academic Societies meeting in April 2025 and at the annual California Collaborative Advocacy Conference in May 2025.

## Materials and methods

Resident Advocacy Rotation

Beginning in July 2021, rotating pediatric faculty facilitated two-hour advocacy workshops through the twice-monthly RAR in partnership with AAP-CA over Zoom. AAP-CA is the organization through which the AAP chapters in California engage in statewide advocacy. Trainees from any of California’s 14 pediatric training programs were invited to register to attend these two-hour workshops. RAR facilitators were faculty from training programs in the collaborative and were encouraged to teach at least two sessions annually. All sessions had representatives from the AAP-CA join, and some sessions included guest speakers from community organizations.

The RAR learning objectives were to (1) understand the roles of pediatricians in advocacy, (2) review basic knowledge of advocacy and the policy process, (3) be able to use an advocacy communication skill to contribute to an active campaign, (4) provide opportunities to advocate for priority child health issues, and (5) promote collaboration with the AAP and child advocacy partner organizations.

Each RAR workshop opens with a basic introduction to the AAP and the role of pediatricians in advocacy, then focuses on a particular child health advocacy topic (e.g., healthcare access, vaccines). Facilitators choose their own topic, keeping in mind the AAP-CA policy priorities for that year. Participants then apply a particular advocacy communication skill to the topic, followed by a group debrief and evaluation. Advocacy communication skills included op-ed writing, social media advocacy, public comment on regulations, media interviews, bill analysis, communicating with policymakers, honing an elevator pitch, letter writing, and narrative storytelling. Prework for each RAR workshop was emailed to participants one week in advance and typically included key articles and resources for the session’s highlighted advocacy topic.

The RAR was administered by one author (SW), who provided facilitators with a standardized RAR slide deck to serve as an outline for their presentation. Facilitators adapted this deck and added content on their selected child health advocacy topic. New facilitators were trained by Dr. Wu, co-facilitated a session with a more experienced facilitator, or both. Facilitators discussed successes and challenges of the RAR at monthly virtual and annual in-person California Collaborative faculty meetings.

Survey administration

Between July 1, 2021, and June 30, 2024, all trainees who participated in RAR were invited to complete a voluntary, anonymous survey in Qualtrics by the facilitator immediately following the session by scanning a QR code or via a link in the webinar chat. We utilized the AAP’s Advocacy Training Guide Evaluation survey [14], which included four questions about their satisfaction with the session on a four-point Likert scale and a single open-ended question, “What part of the training was most valuable to you?” (Appendices).

Faculty who had facilitated RAR sessions or led their institution’s advocacy rotations between July 2021 and January 2024 were invited through email to complete a voluntary, anonymous survey in Qualtrics between January and May 2024. Survey questions were developed by the authors through expert consensus and literature review. Faculty were asked questions about their satisfaction with the session on a five-point Likert scale, open-ended questions about their perspectives on RAR, and a brief demographic questionnaire (Table 1).

**Table 1:** Resident Advocacy Rotation faculty feedback survey.

Survey question	Response
Introduction	The purpose of this anonymous, voluntary survey is to understand your experience as faculty working with pediatric trainees on the AAP Resident Advocacy Rotation (AAP-RAR). All responses are confidential and will be reported in aggregate form
Institution	What institution do you work for?
Part 1: Session facilitators	
Have you taught any Resident Advocacy Rotation sessions?	o Yes o No o Unsure
How many Resident Advocacy Rotation sessions have you taught?	__________________________________________
Please rate how much you agree or disagree with the following statements:	
Teaching trainees in the AAP Resident Advocacy Rotation was a valuable experience for me	5 - Strongly agree 4 - Agree 3 - Neither agree nor disagree 2 - Disagree 1 - Strongly disagree 0 - Not applicable or don’t know
Participating in the AAP Resident Advocacy Rotation was a valuable experience for my institution	5 - Strongly agree 4 - Agree 3 - Neither agree nor disagree 2 - Disagree 1 - Strongly disagree 0 - Not applicable or don’t know
I am interested in teaching the AAP Resident Advocacy Rotation again in the future	5 - Strongly agree 4 - Agree 3 - Neither agree nor disagree 2 - Disagree 1 - Strongly disagree 0 - Not applicable or don’t know
What was most rewarding about teaching in the Resident Advocacy Rotation?	__________________________________________
What was least rewarding about teaching in the Resident Advocacy Rotation?	__________________________________________
What do you feel should change about the Resident Advocacy Rotation?	__________________________________________
How has participating in the Resident Advocacy Rotation impacted your career or professional development, if any?	__________________________________________
Additional feedback	Please share any additional feedback about your experience teaching on the Resident Advocacy Rotation.
Part 2: Rotation leaders	
Are you a Community/Advocacy rotation director or advocacy track leader who arranges for your trainees to attend the Resident Advocacy Rotation?	o Yes o No o Unsure
Please rate how much you agree or disagree with the following statements:	
The AAP Resident Advocacy Rotation is a valuable learning experience for trainees at my institution	5 - Strongly agree 4 - Agree 3 - Neither agree nor disagree 2 - Disagree 1 - Strongly disagree 0 - Not applicable or don’t know
The AAP Resident Advocacy Rotation addresses a gap in my institution’s advocacy/community curriculum	5 - Strongly agree 4 - Agree 3 - Neither agree nor disagree 2 - Disagree 1 - Strongly disagree 0 - Not applicable or don’t know
I want the trainees at my institution to continue to attend the AAP Resident Advocacy Rotation in the future	5 - Strongly agree 4 - Agree 3 - Neither agree nor disagree 2 - Disagree 1 - Strongly disagree 0 - Not applicable or don’t know
What were the best aspects of your trainees participating in the Resident Advocacy Rotation?	__________________________________________
What aspects of the rotation could be improved?	__________________________________________
Additional Feedback	Please share any additional feedback about the Resident Advocacy Rotation.
Part 3: Demographics	
Which of the following best describes your gender identity? (Select one)	o Female o Male o Transgender Female o Transgender Male o Gender non-conforming/non-binary o Prefer to self-describe __________________
Which of the following best describes your racial or ethnic identification? (Select all that apply)	▢ American Indian or Alaska Native ▢ Asian or Asian American ▢ Black or African American ▢ Hispanic, Latino, or of Spanish Origin ▢ Native Hawaiian or Other Pacific Islander ▢ Non-Hispanic, White ▢ Prefer to self-describe __________________ ▢ Prefer not to answer
What is your academic title? (If any)	o Instructor o Assistant Professor o Associate Professor o Professor o No academic title o Other _______________________________
If applicable, how many years out of residency are you?	o 0-5 years o 6-10 years o 11-15 years o 16-20 years o 21-25 years o 26-30 years o 30+ years o Decline to answer o Does not apply

The Institutional Review Board of the University of California, Los Angeles deemed this project exempt from review.

Data analysis

Multiple-choice questions in both the trainee and faculty surveys were analyzed using descriptive statistics, and we report frequencies, percentages, median, and interquartile range for questions rated on a Likert scale. Open-ended questions in both surveys were analyzed using conventional content analysis by all three authors through inductive qualitative methods [15]. Specifically, two authors (VS, CG) read all open-ended responses and independently developed preliminary codes inductively. They reconciled the codes together to develop a codebook before re-coding all survey responses using the agreed-upon codebook in Dedoose. Each survey response received two independent reviews, with any differences discussed and reconciled together. All three authors then categorized codes into themes through discussion and refined the list of themes until consensus was reached. The themes were re-presented to a subset of faculty members at a California Collaborative meeting in May 2025 to verify validity.

## Results

Trainee evaluation

Between July 1, 2021, and June 30, 2024, 67 RAR sessions were held. Overall, 312 out of 721 (43% response rate) registered trainees completed the survey. Registered trainees were from 13 pediatric training institutions in California and represented both university- and community-based training sites: Children’s Hospital Los Angeles, Cottage Children’s Medical Center, Kaiser Permanente Oakland, Kaiser Permanente Los Angeles, Loma Linda University, Naval Medical Center San Diego, Stanford University, University of California Davis, University of California Irvine-Children’s Hospital of Orange County, University of California Los Angeles, University of California San Diego/Rady Children’s Hospital, University of California San Francisco Benioff Children’s Hospital Oakland, and University of California San Francisco Benioff Children’s Hospital San Francisco. Registered trainees included medical students (3.7%), residents PGY1-4 (37.7%, 28.0%, 29.5%, 0.1%, respectively), and fellows (0.4%). Overall, trainees agreed that RAR taught specific advocacy strategies, introduced new ideas, provided useful materials, and included content they hoped to gain (Table 2).

**Table 2:** Trainee (N = 312) satisfaction with the RAR, rated on a four-point Likert scale (4 = strongly agree, 3 = agree, 2 = disagree, 1 = strongly disagree). IQR = interquartile range; RAR = Resident Advocacy Rotation

Trainee satisfaction with rotation, N = 312	n	Strongly agree, n (%)	Agree, n (%)	Disagree, n (%)	Strongly disagree, n (%)	Median (IQR)
I learned specific advocacy strategies that I will use in my advocacy efforts	312	149 (48)	155 (50)	6 (2)	2 (1)	3 (3–4)
The training included new ideas that inspired me to action	312	145 (47)	153 (49)	11 (4)	3 (1)	3 (3–4)
The materials will be useful to me after I leave the training	312	159 (51)	145 (47)	5 (2)	3 (1)	4 (3–4)
The content included things I was hoping to gain in the training	312	149 (48)	154 (49)	5 (2)	4 (1)	3 (3–4)

Qualitative analysis of free text responses answering what aspects of the training were most valuable revealed several themes (Table 3). Participants highlighted the value of learning about advocacy resources, specifically learning from the AAP directly and accessing AAP advocacy resources, ultimately leading to a sense of self-efficacy. Additionally, residents highlighted the value of collaborating with trainees from other programs and learning practical advocacy skills such as bill analysis, communicating with legislators, and op-ed writing. Residents valued gaining specific policy knowledge around the legislative process and policy topics such as mental health and juvenile justice. Lastly, residents appreciated learning from community partners. Trainees identified spending more time on the practical and collaborative aspects of the training as an area for improvement.

**Table 3:** Themes of trainee’s perspectives on the most valuable aspect of the RAR. AAP = American Academy of Pediatrics; RAR = Resident Advocacy Rotation

Theme	Subtheme	Illustrative quotes
Advocacy resources	Learning from the AAP and how to access AAP Advocacy resources	“Learning about the different bills (on the AAP website) and the stance AAP takes on each of them, ultimately assisting with the process of drafting and submitting a letter to the senator(s). I do not personally have much advocacy experience so this training provided great insight into how important it is for me to get involved, on a local or state level, to advocate for my patients in this way.”
	Resident self-efficacy in getting involved in advocacy	“Seeing how possible it was to get involved at a resident level.” “Hearing experiences and background from pediatricians who have been involved in advocacy work.”
Collaboration	The benefits of collaborating amongst trainees	“Discussing the bill as a large group and hearing other people’s perspectives, answering questions/curiosities together.”
	Learning from community experts	“Small group activity to discuss the bill, and listening to the expert (…) discuss the bill.”
Practical advocacy skills	Bill analysis	“I really appreciated walking through a bill and breaking it down into the stakeholder chart because it was a direct application of theoretical advocacy work.”
	Communicating with legislators	“Discussing how to specifically contact senators and advocate for issues important to us.”
	Media training	“Learning specifics about how to engage with media/interview with a reporter and having an opportunity to practice those skills.”
	OpEd	“Coming up with a lede as a group was so helpful!! I learned a lot from my group.”
	Drafting comments on proposed policies	“Learning about the federal proposed rule commenting project, and practice researching and writing a comment.”
	Social Media	“Understanding how easy it is to advocate via social media.”
Acquiring knowledge	Specific policy knowledge (e.g., juvenile justice system, mental health, injury prevention, etc.)	“Learning about the history of juvenile detention centers and the trends throughout time. I also found it interesting and helpful to see juvenile justice system connects to school disciplinary policies.”
	Understanding the legislative process	“Learning about the different steps a law takes to be passed so that I can better understand what is happening to the bills related to child health.”
Opportunities for improvement	Wanting more time for collaboration/group work	“I wish we had spent more time on the end portion rather than the beginning to focus on actually practicing.”

Faculty evaluation

Overall, 17 out of 25 (68% response rate) faculty facilitators completed the survey, representing 10 different pediatric residency programs. Demographics of faculty respondents are described in Table 4. The majority of respondents were female (94%), held an academic title (81%), and were 6+ years after completing residency (81%). Respondents had facilitated an average of 2.5 RAR sessions, with a range of 1-6 sessions. Most faculty facilitators agreed that participating in RAR was a valuable experience for them and their institution (Table 5). The majority of respondents strongly agreed they would be interested in teaching the RAR in the future.

**Table 4:** Demographics of RAR faculty facilitator respondents (N = 16*). *: One faculty respondent declined to complete the demographic survey. RAR = Resident Advocacy Rotation

Demographic characteristic	Number (%)
Gender	
Female	15 (94)
Male	1 (6)
Race/Ethnicity	
Non-Hispanic, White	10 (63)
Asian or Asian American	6 (38)
Academic title	
Instructor	1 (6)
Assistant Professor	4 (25)
Associate Professor	3 (19)
Professor	5 (31)
No academic title or does not apply	3 (19)
Years out of residency	
0–5 years	3 (19)
6–10 years	5 (31)
11–15 years	4 (25)
16+ years	4 (25)

**Table 5:** Faculty (N = 17) and advocacy rotation or track director (N = 11) satisfaction with the RAR, rated on a five-point Likert scale (5 = strongly agree, 4 = agree, 3 = neither agree nor disagree, 2 = disagree, 1 = strongly disagree). *: One respondent rated this statement “Not applicable” and was removed from the analysis for this statement. IQR = interquartile range; RAR = Resident Advocacy Rotation

Faculty facilitator (n = 17) satisfaction	n	Strongly agree, n (%)	Agree, n (%)	Neither agree nor disagree, n (%)	Disagree, n (%)	Strongly disagree, n (%)	Median (IQR)	Advocacy or rotation track director (n=11) satisfaction	n	Strongly agree, n (%)	Agree, n (%)	Neither agree nor disagree, n (%)	Disagree, n (%)	Strongly disagree, n (%)	Median (IQR)
Teaching trainees in the AAP RAR was a valuable experience for me	17	11 (65)	6 (35)	0 (0)	0 (0)	0 (0)	5 (4–5)	The AAP RAR is a valuable learning experience for trainees at my institution	11	9 (82)	2 (18)	0 (0)	0 (0)	0 (0)	5 (5–5)
Participating in the AAP RAR was a valuable experience for my institution	17	11 (65)	3 (18)	3 (18)	0 (0)	0 (0)	5 (4–5)	The AAP RAR addresses a gap in my institution’s advocacy/community curriculum	11	5 (46)	2 (18)	0 (0)	0 (0)	4 (36)	4 (1–5)
I am interested in teaching the AAP RAR again in the future*	16	9 (53)	7 (41)	0 (0)	0 (0)	0 (0)	5 (4–5)	I want the trainees at my institution to continue to attend the AAP RAR in the future	11	10 (91)	1 (9)	0 (0)	0 (0)	0 (0)	5 (5–5)

Overall, 11 of the 17 faculty respondents were their institution’s Community Pediatrics/Advocacy rotation directors. The majority of directors felt RAR was a valuable learning experience for their trainees and would continue to send their trainees in the future (Table 5). However, directors were mixed on whether RAR addressed a gap in their institution’s advocacy curriculum, with some strongly agreeing and others strongly disagreeing.

In open-ended responses, faculty identified their passion for teaching advocacy, collaboration with faculty and trainees across the state, partnership with community organizations to teach trainees, and opportunity to grow their own skills and career as the most rewarding aspects of participating in the RAR (Table 6). They identified six additional subthemes for improvement opportunities: time required for preparation; lack of engagement by some trainees; faculty difficulty with online conferencing software; a desire for more timely real-world advocacy items from the AAP or other groups; a desire for more longitudinal opportunities with trainees rather than a single one-off workshop; and, finally, suggestions for changes to the timing within the two-hour session.

**Table 6:** Themes of faculty perspectives on the RAR. RAR = Resident Advocacy Rotation; AAP = Academy of Pediatrics

Theme	Subtheme	Illustrative quote
Most rewarding aspects of RAR participation	Passion for teaching advocacy	“Sharing information, skills, and experience that I am passionate about so that the next gen[eration] of pediatricians will be equipped to be changemakers for kids!”
	Collaboration with faculty and trainees at institutions across the state	“Watching joint collaboration of residents across programs.”
	Partnering with community organizations to teach	“I’ve appreciated building connections to other policy organizations.”
	Opportunities for faculty’s own career growth and skill development	“I listed it as a ‘regional’ teaching experience that helped me for promotion to associate professor. I also was able to sponsor younger faculty/fellows to co-teach with me to provide them opportunities to teach advocacy as well.”
Opportunities for improvement of RAR	Time for preparation and participation	“Not having protected time to prepare and present.”
	Lack of engagement by some trainees	“Sometimes there’s a lack of preparation or engagement on the part of trainees which makes it difficult to have a discussion.”
	Faculty difficulty with online conferencing software	“Difficulty teaching via Zoom across multiple institutions. Zoom is not my strong suit!”
	Desire for more participation from the American AAP and other real-world advocacy opportunities	“It would be great if there was more timely advocacy action items brought to us by the AAP.”
	Desire for more longitudinal opportunities rather than a single one-off	“Opportunity to create more longitudinal collaboration on an advocacy project or letter writing.”
	Structure/timing of session	“More structure around explaining to residents the impact of the AAP, examples of projects, and roles they could consider for the future.”

## Discussion

In its first three years, RAR was widely viewed as a valuable learning experience by trainees and faculty facilitators. Trainees and faculty emphasized that collaborating with other institutions and community organizations, engaging in advocacy teaching and learning, and developing their skills were especially rewarding aspects of the training. RAR provides one potential model for sharing educational resources through a multi-institutional advocacy collaborative that coordinates and delivers advocacy-focused, synchronous teaching sessions for trainees statewide.

RAR builds upon the work of prior advocacy education initiatives and collaboratives. Studies of the educational impact of regional advocacy education collaboratives on resident learning remain limited [6,8-12]. The New Jersey Pediatric Residency Advocacy Collaborative (NPRAC) developed a multi-institutional “Morning Report” focused on teaching social determinants of health, though its evaluation only included 35 trainees [9]. Similar advocacy-related initiatives in Toronto, Boston, and Louisiana were also more limited in scope or participants [10-12]. Notably, beyond trainee didactics, several collaboratives have been able to implement regionwide advocacy-focused community partnerships and quality improvement projects. For example, NJPRAC launched a program to address community-identified needs during the COVID-19 pandemic [16]. The Carolinas Collaborative implemented food insecurity screening and referral regionwide, which showed increased screening, referral, and coding among their eight teaching clinics [17]. While this RAR evaluation focuses on resident and faculty perceptions of their training, harnessing the collaborative effort to change physician behavior and improve patient outcomes through advocacy is the ultimate goal for residency training in advocacy.

RAR continues to evolve to address areas for improvement raised in evaluations and the changing advocacy education needs of the California Collaborative. Compared to its initial format in 2021, RAR now places even greater emphasis on real-world advocacy opportunities from AAP and community partners, and allocates more time to discussion and advocacy action. RAR coordinators also offer faculty development to increase comfort with session structure and videoconferencing technology. Notably, ACGME recently revised the requirements for advocacy education in pediatric training programs, transitioning to requiring a minimum of 40 weeks of ambulatory experiences with elements of community pediatrics and child advocacy interwoven throughout [4,18]. Given that RAR is offered twice monthly, this change could allow for more longitudinal engagement with a greater proportion of trainees attending multiple RAR sessions.

There are several limitations to this study. The trainee survey response rate was low, likely due to multiple factors. We were not able to track actual attendance and used participant registration as a proxy. Some registered trainees did not attend the session; therefore, the denominator is likely overestimated. In addition, we relied on individual faculty facilitators to provide the trainee survey link at each session and cannot verify that this always occurred. Although sessions followed the same general format based on a core set of template slides and agendas, facilitators had discretion to select their own topics and customize the content. In addition, there was no adjustment for session enrollment; thus, responses may overweigh those sessions with a high number of participants. A small number of trainees participated in more than one session, having the opportunity to complete the evaluation after each session, and we were not able to adjust for multiple responses. Moreover, our faculty facilitators were nearly all women (23/25, 92%), as were faculty demographic survey respondents (15/16, 94%), which may limit the generalizability of these faculty perspectives to more gender-diverse groups of facilitators.

While this study evaluated trainee and faculty satisfaction with RAR, future studies should aim to quantify the impact of advocacy actions taken from RAR sessions, such as the number of legislator letters sent, as well as long-term physician behavior change and patient-related outcomes. Future studies should also examine how broader trends in medical education affect pediatric advocacy education, including ACGME changes and the growing presence of advocacy education in medical school.

## Conclusions

The RAR offers one model for delivering advocacy education across regional pediatric training programs, using a shared statewide curriculum to pool teaching resources among institutions. Over its initial three years, trainees and faculty facilitators reported high satisfaction with the experience, with trainees indicating that sessions provided advocacy strategies they anticipated using in their own efforts. RAR continues to make adjustments in response to trainee and faculty needs and feedback. Future research should focus on the impacts of advocacy efforts undertaken by RAR participants on the health of pediatric patients and communities, as well as perceived impact on the future careers of trainees. While physician advocacy is increasingly emphasized in medical training, resources and expertise to teach advocacy may not be readily available. This work describes one approach to addressing these gaps through regional collaboration among pediatric training programs, an approach that trainees and faculty found valuable.

## Appendices

Trainee feedback survey

**Table 7:** Trainee feedback survey, adapted from the American Academy of Pediatrics’ Advocacy Training Modules Evaluation Survey.

Survey question	Rating (1–4)
I learned specific advocacy strategies that I will use in my advocacy efforts:	4 - Strongly agree 3 - Agree 2 - Disagree 1 - Strongly disagree
The training included new ideas that inspired me to action:	4 - Strongly agree 3 - Agree 2 - Disagree 1 - Strongly disagree
The materials will be useful to me after I leave the training:	4 - Strongly agree 3 - Agree 2 - Disagree 1 - Strongly disagree
The content included things I was hoping to gain in the training:	4 - Strongly agree 3 - Agree 2 - Disagree 1 - Strongly disagree
What part of the training was most valuable to you?	________________________________________________
